# Towards improved health service quality in Tanzania: appropriateness of an electronic tool to assess quality of primary healthcare

**DOI:** 10.1186/s12913-019-3908-5

**Published:** 2019-01-22

**Authors:** Sabine Renggli, Iddy Mayumana, Dominick Mboya, Christopher Charles, Christopher Mshana, Flora Kessy, Tracy R. Glass, Constanze Pfeiffer, Alexander Schulze, Ann Aerts, Christian Lengeler

**Affiliations:** 1 Department of Epidemiology and Public Health, Swiss Tropical and Public Health Institute, P.O. Box, 4002 Basel, Switzerland; 2 University of Basel, Basel, Switzerland; 3 Ifakara Health Institute, Dar es Salaam/Ifakara, United Republic of Tanzania; 4 Swiss Agency for Development and Cooperation, Berne, Switzerland; 5 Novartis Foundation, Basel, Switzerland

**Keywords:** Quality of care, quality assessment tool, Tanzania, electronic tool, supportive supervision, universal health coverage

## Abstract

**Background:**

Progress in health service quality is vital to reach the target of Universal Health Coverage. However, in order to improve quality, it must be measured, and the assessment results must be actionable. We analyzed an electronic tool, which was developed to assess and monitor the quality of primary healthcare in Tanzania in the context of routine supportive supervision. The electronic assessment tool focused on areas in which improvements are most effective in order to suit its purpose of routinely steering improvement measures at local level.

**Methods:**

Due to the lack of standards regarding how to best measure quality of care, we used a range of different quantitative and qualitative methods to investigate the appropriateness of the quality assessment tool. The quantitative methods included descriptive statistics, linear regression models, and factor analysis; the qualitative methods in-depth interviews and observations.

**Results:**

Quantitative and qualitative results were overlapping and consistent. Robustness checks confirmed the tool’s ability to assign scores to health facilities and revealed the usefulness of grouping indicators into different quality dimensions. Focusing the quality assessment on processes and structural adequacy of healthcare was an appropriate approach for the assessment’s intended purpose, and a unique key feature of the electronic assessment tool. The findings underpinned the accuracy of the assessment tool to measure and monitor quality of primary healthcare for the purpose of routinely steering improvement measures at local level. This was true for different level and owner categories of primary healthcare facilities in Tanzania.

**Conclusion:**

The electronic assessment tool demonstrated a feasible option for routine quality measures of primary healthcare in Tanzania. The findings, combined with the more operational results of companion papers, created a solid foundation for an approach that could lastingly improve services for patients attending primary healthcare. However, the results also revealed that the use of the electronic assessment tool outside its intended purpose, for example for performance-based payment schemes, accreditation and other systematic evaluations of healthcare quality, should be considered carefully because of the risk of bias, adverse effects and corruption.

## Background

A core part of Universal Health Coverage (UHC) is access to essential health services of sufficient quality to be effective [1]. To assess health service coverage the UHC monitoring framework uses the concept of effective coverage [2]. Effective coverage is given when people who need health services obtain them in a timely manner and at a level of quality that allows achieving the desired effects [3]. Thus, effective coverage combines intervention need, use and quality. It stands in contrast to crude coverage, which only focuses on intervention access or use [4]. Consequently, to reach effective coverage and therewith the target of UHC, it is vital to address the issue of quality of healthcare. To do so, quality of healthcare must be assessed and monitored, and the results have to be actionable. However, data on quality of healthcare in low- and middle-income countries (LMICs) is hardly available [5–7]. One reason for this is the focus in the past on increasing access and use rather than on providing high-quality services [7]. Additionally, quality of care is much more difficult to assess routinely, and no agreed means to monitoring quality exist [8–10]. Current quality measures are insufficiently validated and not implemented consistently, making it hard to compare between settings [5, 7, 11, 12].

**Fig. 1:**
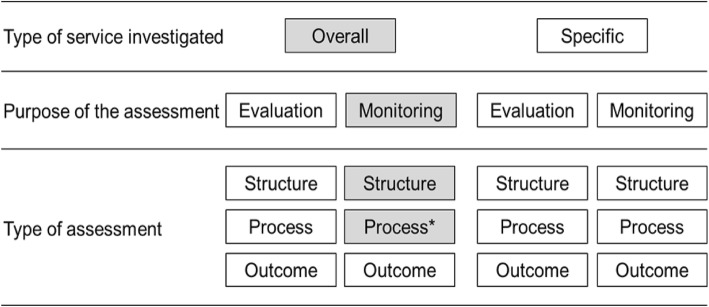
Design options of healthcare quality assessment tools. Shaded in grey the design of the e-TIQH assessment tool; asterisk indicates the uniqueness of the e-TIQH assessment tool

Generally, the design of healthcare quality measurements is given by the service whose quality is being investigated as well as the purpose and the type of assessment (Fig. 1) [13].

Quality assessment tools found in literature either look at overall quality of care or focus on more specific services (for example on HIV/AIDS). Some tools primarily aim to systematically evaluate service quality with the purpose of providing evidence for national policy, planning or management decisions, or for accreditation and licensing [13–20]. When examining overall quality of care, such assessment tools tend to be lengthy, time-consuming and technically demanding [13]. In contrast, other tools mainly intend to routinely monitor service quality with the purpose to either report on progress made or steer improvement measures at local level [13, 15, 16].

In terms of quality measurement type, Donabedian proposed to distinguish between structure, process and outcome assessments [21]. Outcome assessments measure the medical outcomes of care, but their usefulness is limited due to the attribution gap between quality of care and outcomes [13, 21]. Thus, process assessments, which examine the process of care delivery itself, might be more relevant regarding whether healthcare is properly practiced [21–24]. Lastly, structure assessments refer to the setting in which healthcare takes place [21]. However, also here a direct link between increased structural quality and better health outcomes is weak [21, 25–27]. This suggests that quality of care is more effectively improved when targeting process elements [27–29]. Concretely, this means that for quality assessment tools, which primarily aim to routinely steer improvement measures, it might be most effective to focus on processes and structural key indicators, which assess whether structures are of sufficient quality (adequacy). Focusing on healthcare processes would also be in-line with what was proposed as an approach for measuring effective coverage [4]. This as well implies that such assessment tools would not need to be fully comprehensive to accurately fulfill their purpose, making it more feasible for routine measures in resource constraint settings. However, so far monitoring overall quality of care mainly focused on the structural part of quality by examining the existence of structures (availability) and leaving adequacy under-explored [5, 13, 15, 16, 30–33]. Assessment tools monitoring specific services usually use an approach combining structural and process elements [13, 15, 16, 29, 30]. Yet, it is important to look beyond a single service area to assess primary healthcare more generally in a harmonized holistic way [13].

**Fig. 2:**
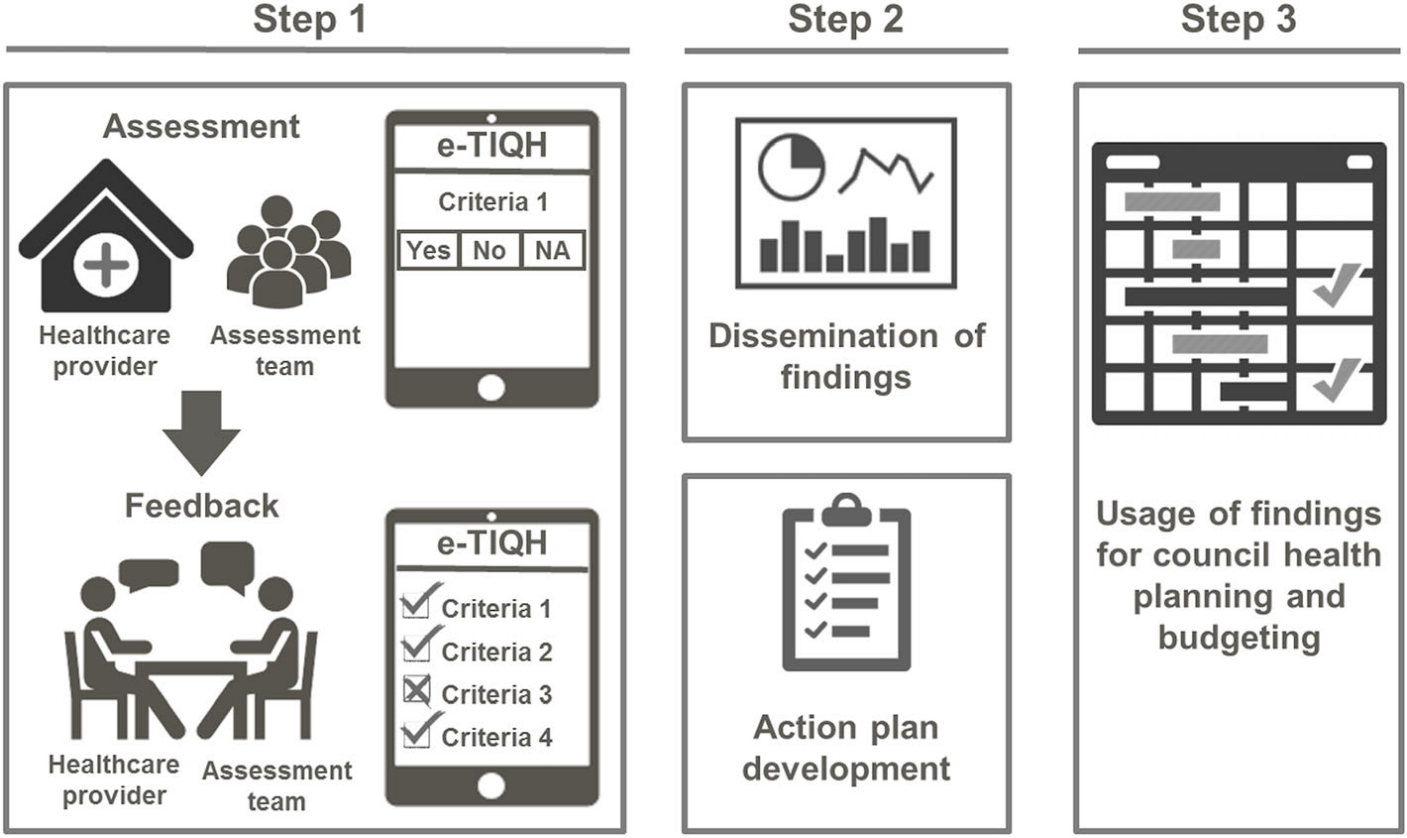
Chart of the three-stage process of the e-TIQH supportive supervision approach (figure previously published in [36])

Apart from assessment tools developed for specific services, there is, to the best of our knowledge, hardly any documentation about quality assessment tools in LMICs that focus on processes and structural adequacy of healthcare with the purpose of routinely steering improvement measures. To fill this gap, we systematically evaluated an approach developed in Tanzania as part of the “Initiative to Strengthen Affordability and Quality of Healthcare”. The aim of the approach was to improve quality of primary healthcare through strengthening routine supportive supervision of healthcare providers, as conducted by Council Health Management Teams (CHMTs). In a first step a systematic assessment of quality of primary care was carried out in out-patient departments of all health facilities within a given council, using the “electronic Tool to Improve Quality of Healthcare (e-TIQH)” (Fig. 2).

The assessment was always concluded with an immediate constructive feedback to the healthcare providers, and joint discussions about how to address the identified quality gaps. In a second step, the findings were discussed at council level with all relevant stakeholders, providing important inputs for the third step, the annual council health planning and budgeting process. The supportive supervision approach and in particular the e-TIQH assessment tool with its indicators have been described by Mboya et al. [34]. This paper now aims to examine how well the e-TIQH assessment tool measures and monitors quality of care. Given the lack of a gold standard regarding how to best measure quality of care, we tried to verify the validity of the e-TIQH assessment tool by using a range of methods. Companion papers will further investigate if the e-TIQH approach contributed to improvements in quality of care and how the approach was able to strengthen routine CHMT supportive supervision [35, 36].

## Methods

### Measurement of quality of care

**Fig. 3:**
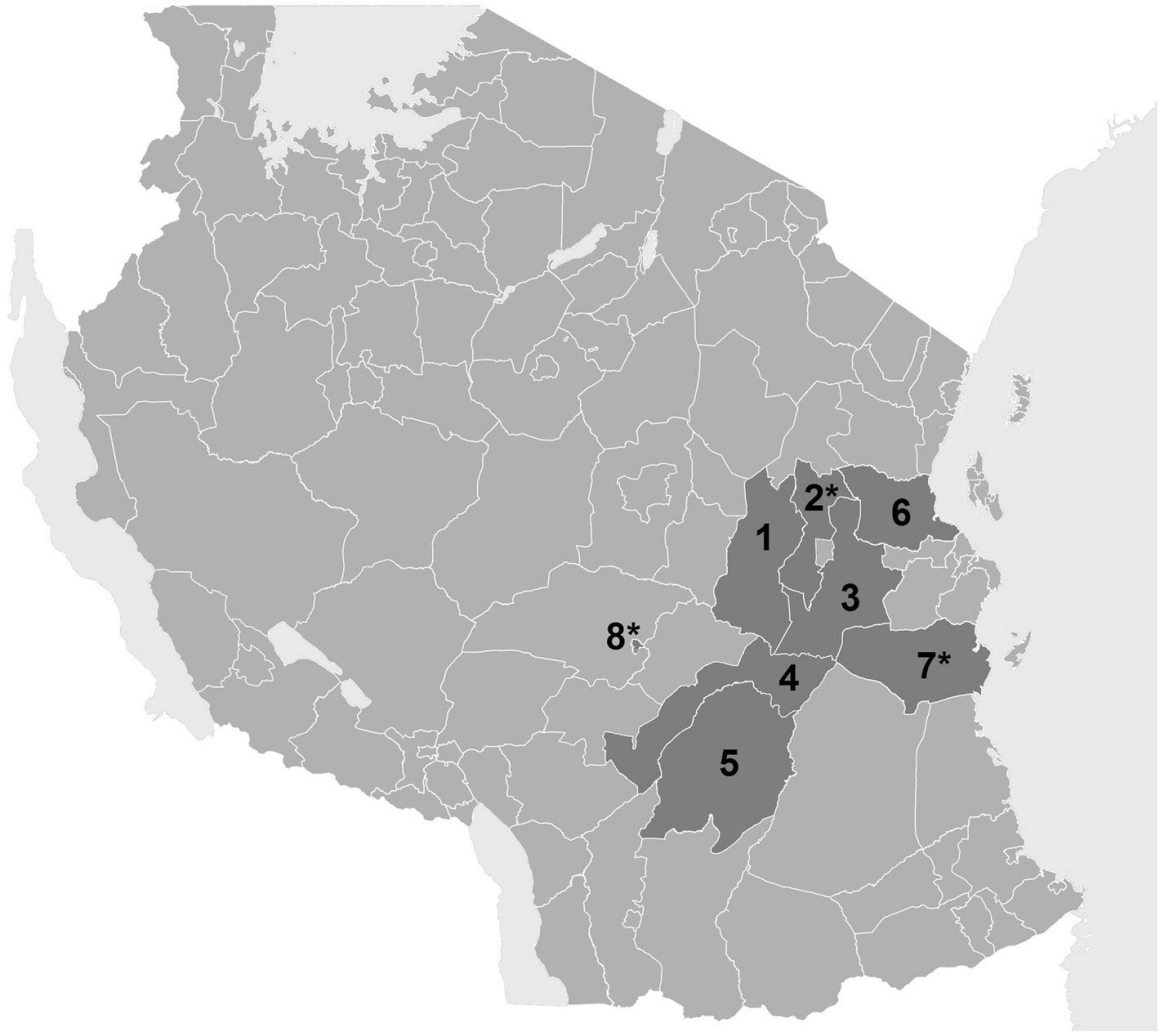
Map of Tanzania with councils where the e-TIQH supportive supervision approach was implemented (status 2008). Morogoro Region: (1) Kilosa DC (later split into Kilosa and Gairo DC), (2) Mvomero DC, (3) Morogoro DC, (4) Kilombero DC, (5) Ulanga DC; Pwani Region: (6) Bagamoyo DC, (7) Rufiji DC; Iringa Region: (8) Iringa MC. Asterisks mark councils selected for qualitative data collection (figure previously published in [36])

Quality of primary healthcare was measured between 2008 and 2014 in out-patient departments of health facilities in up to eight Tanzanian district and municipal councils (DCs and MCs) (Fig. 3).

The list of e-TIQH assessment indicators used to measure primary healthcare was developed in an iterative process and in consultation with key stakeholders, including clinical experts and government representatives. The process strictly followed existing national treatment, supportive supervision, and other guidelines [34]. During the same development process indicators were also grouped into six quality dimensions (QDs): (1) Physical environment and equipment; (2) Job expectations; (3) Professional knowledge, skills and ethics; (4) Management and administration; (5) Staff motivation; (6) Client satisfaction. QD 3 was further divided into four sub-dimensions, making the total number of sections nine. Additionally, indicator weights ranging from 1 (least important) to 5 (most important) were assigned according to their importance for quality of care relative to the other indicators. Points were given for each indicator met, and percentage scores of total possible points were calculated per QD. The score of each QD equally contributed to the overall health facility score. More details regarding score calculations can be found in Mboya et al. [34].

**Fig. 4:**
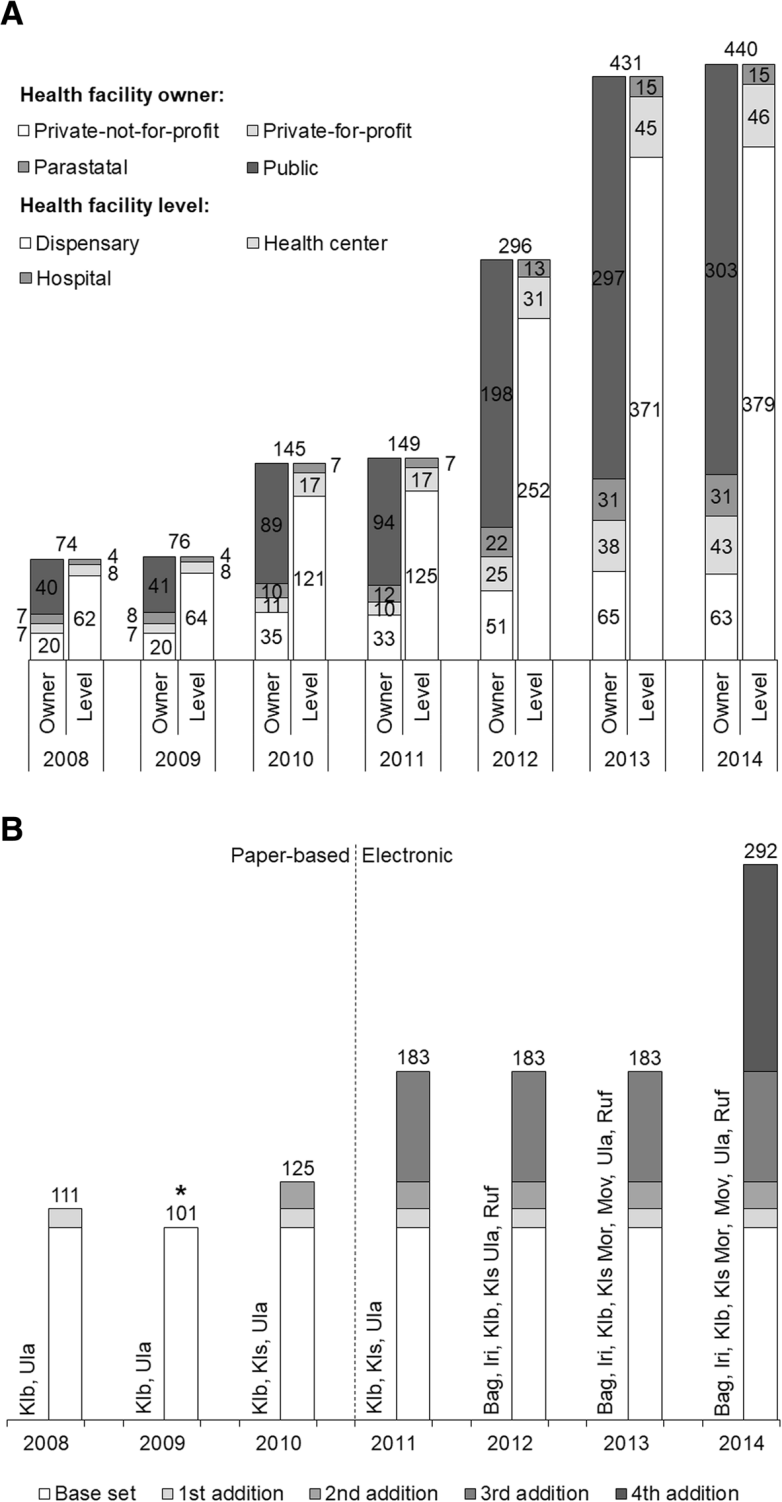
Number of health facilities included in the analysis in each year across all selected councils, by health facility owner and level category (status 2014) (**a**); number of indicators included in the analysis across years and councils (**b**). Bag = Bagamoyo DC, Iri = Iringa MC, Klb = Kilombero DC, Kls = Kilosa DC (later split into Kilosa and Gairo DC), Mor = Morogoro DC, Mvo = Mvomero DC, Ula = Ulanga DC, Ruf = Rufiji DC (status 2008); * Missing indicators due to data entry problems

Data collection between 2008 and 2010 was paper-based, whereas from 2011 onwards this was done electronically using the e-TIQH assessment tool [34]. Due to a phased introduction of the e-TIQH approach and the quality of manually entered data, the number of councils, health facilities and indicators included in the analysis varied between years (Fig. 4). In Fig. 4a health facilities assessed each were categorized based on their owner category (private-not-for-profit, private-for-profit, parastatal, public). In the same figure health facilities were additionally differentiated according to their level of care, with the lowest level being dispensaries, followed by health centers and hospitals. Health centers and hospitals may also have in-patient departments, but only out-patient departments were assessed. Figure 4b illustrates the number of indicators included in the analysis across years and councils.

**Fig. 5:**
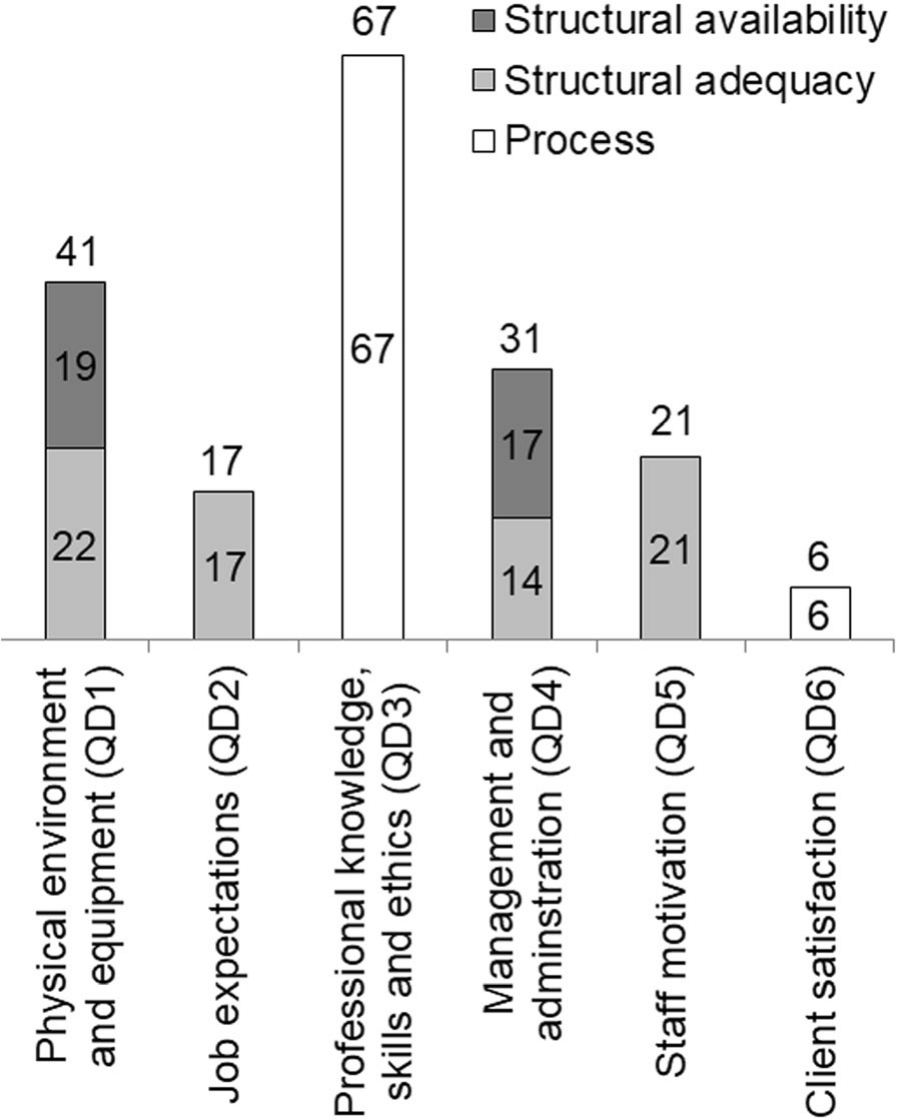
Number of indicators assessed in each quality dimension (QD) by indicator type for the 183 indicator set (Fig. 4b)

The assessment methods included checklists, structured interviews and clinical observations in order to assess processes and structural key indicators primarily focusing on adequacy. For example, a medical doctor would observe whether the healthcare provider adheres to the principles of Focused Antenatal Care during the assessment and management of a pregnant women. To do so, the medical doctor used a checklist, which was developed in-line with national guidelines. Figure 5 illustrates the number of indicators assessed in each QD, according to the indicator type based on Donabedian’s categories (structure, process, outcome) [21].

### Assessing the appropriateness of the e-TIQH assessment tool to measure quality of care

Various methods were triangulated to assess the appropriateness of the e-TIQH assessment tool. First, we explored whether quantitative data obtained from the e-TIQH assessments and qualitatively collected perceptions of quality of healthcare were consistent for different level and owner categories. To do so, we used linear regression models and data from in-depth interviews. The latter was complemented by observational data and personal communication. We also analyzed whether a rank qualitatively assigned to health facilities visited was comparable with the rank achieved according to the quantitative e-TIQH assessment. Additionally, to assess the robustness of the e-TIQH assessment tool we investigated the change in health facility score and rank upon changing the number of indicators (Fig. 4b) and erasing the indicator weights. Finally, we assessed the usefulness of grouping the indicators into the nine QDs and sub-QDs through conducting a confirmatory factor analysis. This was to test whether the factors identified by the factor analysis represent the QDs determined during the development process of the e-TIQH assessment tool. All methods used are hereafter explained in detail.

#### Linear regression model

Mixed linear regression models were developed to look at differences between QDs by health facility level and owner categories. For this only the electronically gathered data between 2011 and 2014 with 183 indicators was used due to several inconsistencies in the manually entered data. Models were derived for the overall score and the six QD scores. Year, health facility level and owner were categorical variables. The variable council was set as a random effect. Third and second order interaction terms were included and then stepwise excluded using Wald tests, whereby the variable with the highest order and *p*-value was excluded first. To confirm model selection the Akaike Information Criterion (AIC), which is an alternative to significance testing for model comparison, was calculated as well. Additionally, a sensitivity analysis was done comparing the random effect model with a fixed effect model using the robust variance estimator.

#### In-depth interviews

In total 24 interviews at council and health facility level were conducted in three councils (Fig. 3). To compare health facility level and owner categories, only the 12 interviews done at council level were included into the present analysis. There we probed for possible differences in quality of care amongst different health facility level and owner categories. Interview partners were sampled purposefully. At council level we interviewed two CHMT members (including co-opted members) as representatives of the public sector. Also, two members of the Council Health Service Board (CHSB), which is the governance body responsible for adequate service delivery and CHMT oversight, were chosen to represent the non-public sector [37]. Interviews were conducted in the first quarter of 2016 by a Swahili speaking female Swiss (SR) and a male native Tanzanian of middle age (IM). From all respondents written informed consent was obtained. Interviews were tape-recorded and transcribed by two native Tanzanian research assistants without being translated into English. The transcripts were managed and coded with MAXQDA software. Data were analyzed using the framework method described by Gale et al. [38], which uses a structured matrix output to systematically reduce and analyze qualitative data. Citations stated in the present manuscript were translated into English by SR and proofread by IM. Further details about the collection and analysis of the in-depth interview data can be found elsewhere [35].

#### Qualitative ranking based on observations

For qualitative data collection a total of six public dispensaries across three councils were visited (Fig. 3) [35]. Based on the information collected, the researchers (SR, IM) individually ranked the public dispensaries according to their personal subjective impression about overall quality of care. To do so they took into account the six e-TIQH QDs, about which they had an in-depth knowledge due to extensive preparational work prior to the onset of the qualitative data collection. Afterwards, they discussed their ranking and agreed to one common ranking. This purely qualitative ranking was then compared with the rank dispensaries had achieved according to the quantitative e-TIQH assessment in order to investigate consistency of the quantitative and qualitative data.

#### Number and weights of indicators

To compare indicator sets consisting of different numbers of indicators (Fig. 4b), 2014 overall health facility scores based on unweighted indicators were calculated for various indicator sets and ranked. For each health facility the positive difference in score and rank between the biggest indicator set (292) and each of the smaller in Fig. 4b described sets was calculated. The differences were then averaged across all health facilities to get the average difference in health facility score and rank. The same calculations were done to compare 2014 overall health facility scores and ranks of the 183 indicator set originating once from weighted and once from unweighted indicators.

#### Factor analysis

A factor analysis was performed with the 2014 score of 183 unweighted indicators of each health facility. The distribution of the indicators across the nine factors explaining the biggest variance was examined, in-line with the nine sections of the e-TIQH assessment tool. Each indicator was allocated to the factor to which it showed the strongest association (highest factor loading). Factor loadings range between − 1 and 1 with a strong positive or negative association indicated by loadings close to 1 or − 1, and a weak association with loadings close to 0. Indicators with weak association to the factor to which they were assigned to (factor loadings between − 0.4 and 0.4) were marked because they are unlikely to be relevant for predicting quality of care [39]. Additionally, indicators were defined to be cross loaded if any of the other factor loadings was within a range of 0.2, meaning that these indicators had no clear association to one specific factor [40].

## Results

### Linear regression model

**Fig. 6:**
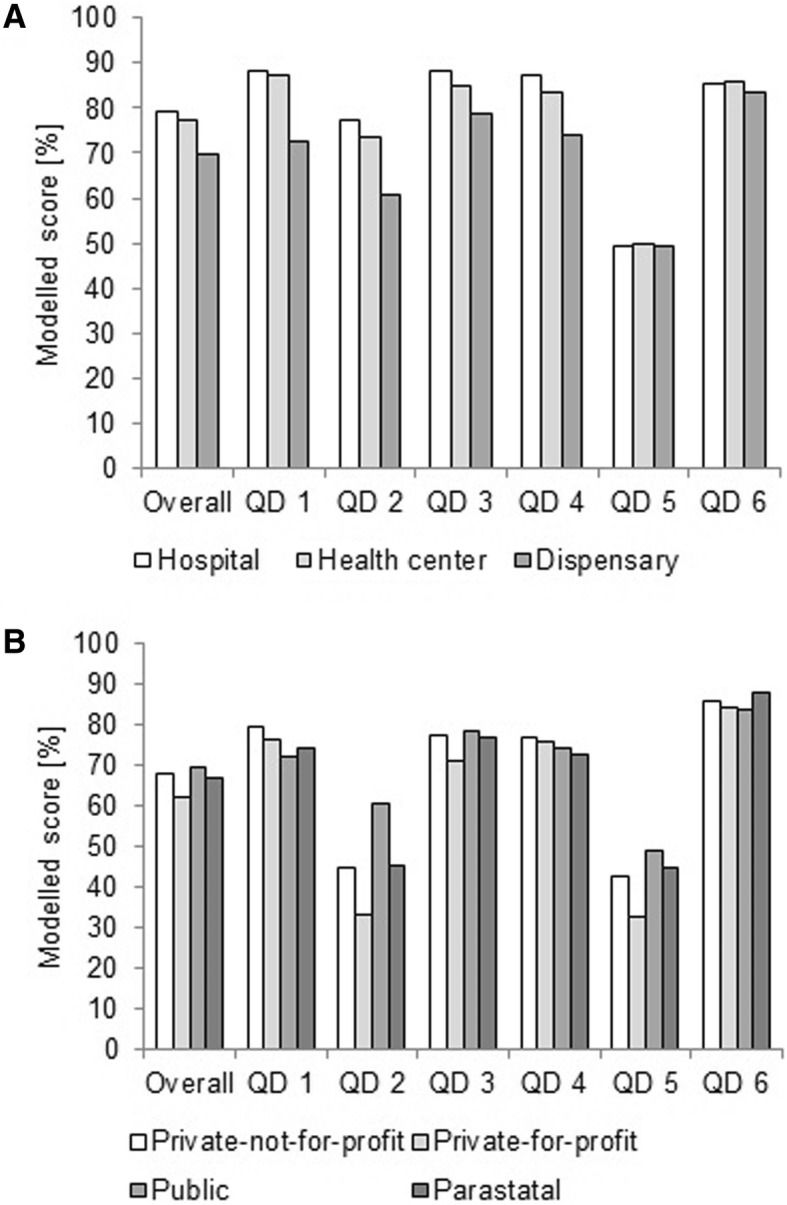
Performance of health facility levels (**a**) and owners (**b**) for the year 2014. In **a** the performance scores for public health facilities only and in **b** for dispensaries only are shown

There was a clear improvement in scores from 2011 until 2014 (Table 1). Health centers and hospitals had a significantly better score compared to dispensaries, except for QD 5 and 6. Apart from QD 6, scores varied amongst owners. Public health facilities had a better overall score than private-not-for-profit, and private-for-profit entities performed significantly worse. For illustrative purposes, performance of health facility levels and owners for the year 2014 is shown graphically in Fig. 6.

**Table 1:** Differences in average overall and quality dimension (QD) scores, expressed as percentages of maximum achievable scores, according to year, health facility level and owner category, while the variable council was set as a random effect

Variable	Overall score	QD 1	QD 2	QD 3	QD 4	QD 5	QD 6
Year (Reference category = 2011)							
2012	3.1 **	−2.7 *	1.2	−1.2	6.1 ***	10.5 ***	2.0
2013	6.5 ***	−0.4	5.8 **	2.7	7.0 ***	15.9 ***	5.4 ***
2014	8.4 ***	4.3 **	4.2 *	6.5 ***	10.2 ***	14.8 ***	7.4 ***
Health facility level (Reference category = Health center)							
Hospital	1.8	1.1	3.7	3.3	3.7	−0.4	−0.5
Dispensary	−7.7 ***	−14.8 ***	−13.2 ***	−6.2 ***	−9.3 ***	−0.5	−2.2
Health facility owner (Reference category = Private-not-for-profit)							
Private-for-profit	−5.5 ***	−3.1*	−11.8 ***	−6.3 ***	−1.2	−9.8 ***	− 1.3
Public	1.8 *	−7.5 ***	15.4 ***	1.2	−2.8 **	6.6 ***	−2.1
Parastatal	−0.9	−5.7 **	0.5	−0.4	−4.3 **	2.0	2.5
Constant	67.3 ***	90.3 ***	54.1 ***	77.0 ***	76.1 ***	28.4 ***	80.5 ***

Asterisks refer to *p*-values indicating the significance of a coefficient * < 0.05, ** < 0.01, *** < 0.001

For all models a large fraction of unexplained variance was attributed to the random effect (data not shown), meaning that scores were strongly correlated within councils

QD 1 = Physical environment and equipment; QD 2 = Job expectations; QD 3 = Professional knowledge, skills and ethics; QD 4 = Management and administration; QD 5 = Staff motivation; QD 6 = Client satisfaction

Models without any interaction terms performed best both according to Wald tests and the AIC. This means trends were the same independent of health facility level and owner category. The sensitivity analysis also showed no major difference between the random effect model and a fixed effect model using the robust variance estimator.

### In-depth interviews

Interviews generally pointed out issues with guideline availability (captured in QD 2), staffing levels and medicine availability (QD 4), staff benefits and rewards (QD 5), as well as with health financing mechanisms (not measured by the assessment tool). The following sections will explore the consistency of the qualitative in-depth interview data with the findings of the regression model described above regarding health facility level and owner categories.

#### Differences between health facility levels

When asking about reasons for differences in healthcare quality at various levels of care, most respondents (9 out of 12) were able to provide information. They pointed out that at higher level of care, meaning at health centers and hospitals, more services were provided (6 of the 9 above mentioned) and there was more and better qualified staff (7/9). For example, a CHMT member said:

*“Most of the skilled personnel can be found at hospital [and] health center level, [which is] different from the dispensary level. But the district [council] medical officer takes into account the different types of services provided at these facilities (…) This means it’s necessary to have nurses and doctors who can provide these services (…) Therefore at dispensary level you cannot find a highly skilled nurse.” (Mvomero DC, CHMT member)*

It was further mentioned that at higher level of care infrastructure (4/9), equipment (4/9) and medicines (1/9) were superior in terms of quantity, quality and type. Respondents also reported that in the light of limited resources, councils tended to prioritize higher level of care (5/9) and non-governmental stakeholders were more likely to support higher-level health facilities (1/9). Some of the here raised issues could be seen as given by the health facility’s mandate, which defines the type of care supposed to be delivered at each level. However, the different mandates had been accounted for when designing the e-TIQH assessment tool through making certain indicators not applicable for lower level of care. Consequently, the fact that the qualitative findings stated here were still in line with what was seen in Table 1 suggested that dispensaries executed their mandate worse than institutions of higher level of care.

#### Differences between health facility owners

Most of the respondents could elaborate reasons for differences in quality of care between the public and private-not-for-profit (11/12) or private-for-profit (10/12) sector. They stated that the private sector performed better in terms of physical environment (private-not-for-profit: 5/11, private-for-profit: 2/10) and availability of equipment (3/11, 2/10), supporting the above findings form QD 1 (Table 1).

According to the respondents, chances to receive guidelines were lower for the private sector (3/11, 3/10), and the private sector was less likely to provide job descriptions and contracts (1/11, 1/10). These perceptions explained the weaker performance of the private sector for QD 2 in Table 1.

Respondents further pointed out that staff working in the private sector were more welcoming and politer than in the public sector (5/11, 2/10), which was captured in QD 3. This was brought up more often for the private-not-for-profit sector, where it was frequently stated in connection with the staff’s intrinsic motivation due to their belief in God (4/11). The issue raised the most was that of unqualified, not well-trained or retired staff working in the private sector (7/11, 7/10). This was mainly affecting scores in QD 3, counteracting the mentioned advantages of the private sector in the same QD. The perceived cause for the problem was the lack of financial resources to employ better qualified staff and the brain drain from the private to the public sector due to better staff benefits in the latter. This was illustrated by a CHSB member as follows:

*“They [faith-based organizations] make the staff… to be tolerant, but in all matters, meaning even for benefits they end up getting paid little (…) this means that they [faith-based organizations] will be looking for a person whose… education level is very low (…) A person like this… you cannot send to a training (…). [Because] the council… will tell you what kind of person they need [when conducting trainings]… you [then] realize you don’t have such a person, that’s why you don’t send him/her. If you don’t send him/her you cannot get the guidelines because to get them you have to go and study” (Mvomero DC, CHSB member)*

In addition, it was raised in some cases that facility in-charges in the private-for-profit sector were not following guidelines (2/10) and tended to over-prescribe medicines to make more profit (4/10). Adding all this together, these statements can well explain the differences in QD 3 between owner categories in Table 1.

Respondents also mentioned the topic of better medicine availability in the private sector (5/11, 3/10), which influenced performance in QD 4, where about half of the measured indicators concerned medicine availability. Thus, issues which were only reflected by one indicator in QD 4, like weaker data reporting by private sector providers (4/11, 4/10) and less frequent routine supportive supervision in private sector health facilities (2/11, 2/10), could not compensate for the substantial bigger problem of medicine availability in the public sector compared to the private sector (Table 1).

Additionally, in the private sector staff was less likely to receive trainings (3/11, 2/10), payment was lower and less timely (4/11, 2/10), and staff benefits and rewards were poorer (4/11, 3/10), which was relevant for the weaker score of private sector providers in QD 5 (Table 1). Respondents further reported a lack of collaboration between private sector providers and council authorities but mentioned that private-not-for-profit facilities were less affected (3/10). This could explain the better performance of private-not-for-profit facilities in QD 5 compared to private-for-profit facilities (Table 1). The fact that across all councils the public sector collaborated with the private-not-for-profit facilities through Private Public Partnerships (PPPs) (7/11), but not with private-for-profit facilities (1/10), further supported this observation. PPPs included the allocation of public employees to the private-not-for-profit sector in exchange for subsidization of certain services or financial support for bigger non-profit facilities. In this regard a member of the CHMT said:

*“I can say… we often work together with them [the faith-based health facilities] […] to some of them we have given personnel… and [in return] they… have been providing some of the services … for example mother and child [health services for] free… But for those… fully private [private-for-profit facilities] I haven’t seen that we have worked with them. There is not something like entering into a contract with them [saying] that you provide services in this area and we give you personnel for that area or we support you here [in this area]…” (Mvomero DC, CHMT member)*

Finally, private-not-for-profit facilities also often got external support from their home institution or faith-based organizations in terms of training, medical products or financial resources (4/11).

### Qualitative versus quantitative ranking

Table 2 shows quantitatively and qualitatively assigned ranks of dispensaries visited. Qualitatively assigned ranks of both researchers were exactly the same and thus no discussions on the common qualitatively assigned rank was required. However, the quantitatively and qualitatively assigned ranks did not completely overlap. This may be explained by the fact that the more services a health facility offered, the more indicators were applicable and thus the more difficult it was to get the full overall score. Secondly, answering an indicator more than once, which was possible for some QDs, made it less likely to obtain the full score for this indicator [34]. These observations suggested that a high number of indicators assessed and/or a high average of answers per indicator led to an underestimation of the health facility score. Thus, this could explain why health facility B and D have a better quantitative rank than A and C.

**Table 2:** Comparison of qualitative and quantitative rank of six public dispensaries

Council	Dispensary	Quali- tative rank	Quanti- tative rank	Quanti- tative score	Number of indicators assessed	Average answers per indicator assessed
1	A	1	3	76%	147	1.79
1	B	2	1	83%	125	1.64
2	C	3	4	66%	163	1.85
3	D	4	2	79%	127	1.49
3	E	5	5	57%	136	1.36
2	F	6	6	52%	152	1.51

### Number and weights of indicators

**Fig. 7:**
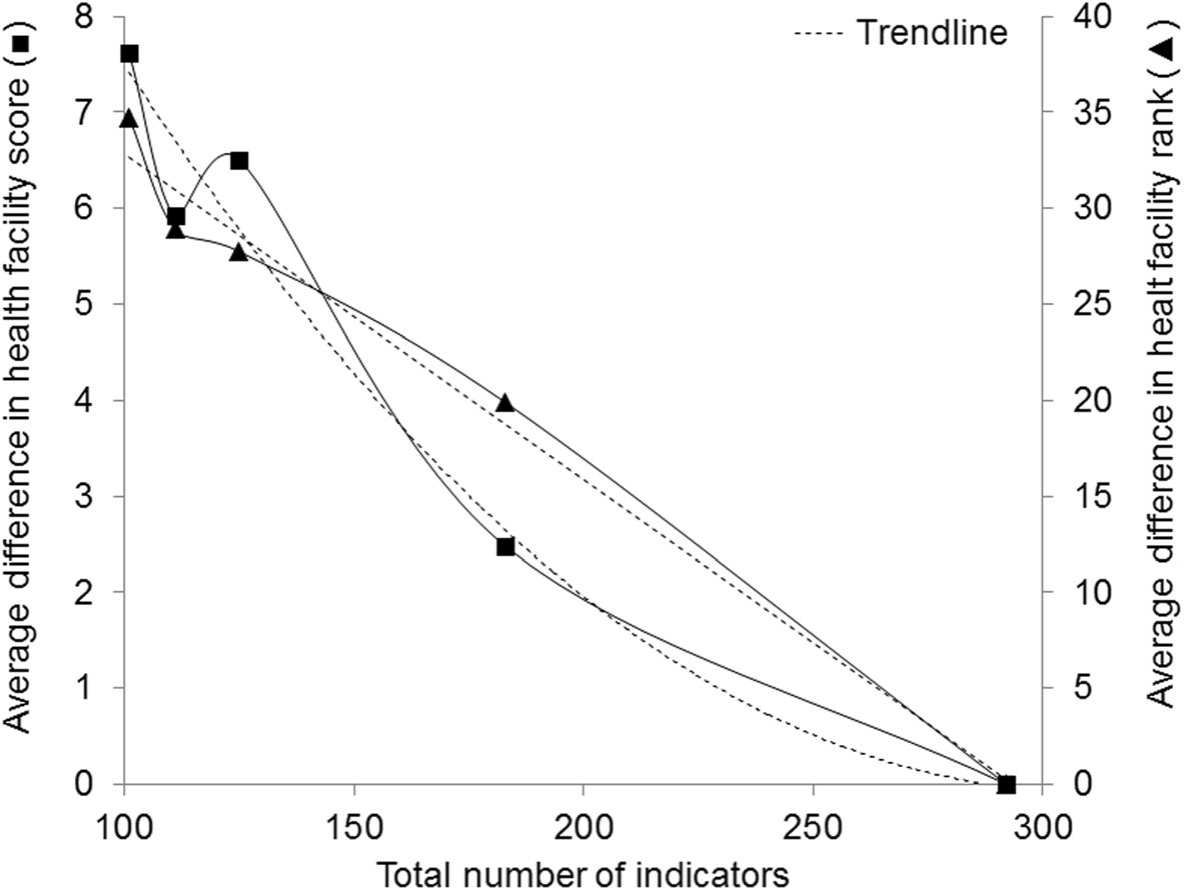
Average difference in 2014 health facility score and rank as a function of the total number of indicators assessed (the score with the largest number of indicators serving as reference). Approximating trend line for average difference in health facility score as a function of total number of indicators assessed is 2nd order polynomial, while for average difference in health facility rank it is linear

Results showed that scores of a given health facility in 2014 were lower for bigger indicator sets, reflecting that it was more difficult to fulfill many indicators compared to fewer (data not shown). Looking at Fig. 7, the average difference in health facility score dropped at the beginning, whereas the line got flatter towards the end. This means that for every additional indicator the average difference in health facility score became smaller, indicating that adding an indicator to a larger number of previous indicators had less influence on the health facility score than adding an indicator to a smaller number of indicators. For difference in rank there was almost a linear decrease, meaning that for each additional indicator the difference in rank stayed the same.

Weighting led to a slightly higher average overall health facility score in 2014 (69.1% vs. 68.4%). This means, indicators with high weights were a little more likely to be answered with “yes” than those with low weights (although the respondents did not know the scores). With an average difference in health facility score of 0.87 and health facility rank of 8.13, the impact of weighting on the overall score and rank was however small compared to the impact of changing the number of indicators.

### Factor analysis

Table 3 shows that the factor to which a particular indicator was allocated to by factor analysis represented for 132 of 183 indicators (72%) the QD to which the same indicator was assigned to during the development process of the e-TIQH assessment tool. Out of these 132 indicators 78% had a strong association to the factor they were assigned to (factor loading 0.4 or more) and only 24% had a similar strong association to another factor (cross-loading within a range of 0.2). This suggested a reliable allocation of these indicators to their respective factors. In contrast, the remaining 51 indicators were allocated differently by factor analysis and during the e-TIQH development process. Of the 51, 73% showed a weak association to the factor they were assigned to (only 27% with factor loading of 0.4 or more) and 88% had a similarly strong association to another factor. In other words, for the e-TIQH assessment tool they seemed to be less relevant for measuring quality of care and were allocated with uncertainty to the corresponding factor. Apart from QD 4, each QD or sub-QD was clearly represented by one factor. For QD 4 most indicators measuring medicine availability (69%) were captured in factor 7, whereas the others were spread across several factors. QD 6 had the highest proportion of indicators with a similar strong association to another factor (83% with cross loading) and a weak association to the factor they were assigned (67% with factor loading below 0.4) relative to the total number of indicators.

**Table 3:** Comparison of indicator allocation between factor analysis and e-TIQH quality dimensions (QDs) defined during the development process of the e-TIQH assessment tool

Factor	e-TIQH QDs^a^	Number of indicators assigned to the same QD^b^			Number of indicators not assigned to the same QD		
		…with cross loading^c^		… with factor loading above 0.4^c^	…with cross loading^d^		… with factor loading above 0.4^d^
1	QD 3B (19)		19 (100%)			8	
		0 (0%)		19 (100%)	8 (100%)		1 (13%)
2	QD 3A (17)		17 (100%)			0	
		0 (0%)		17 (100%)	0		0
3	QD 3D (12)		12 (100%)			4	
		0 (0%)		12 (100%)	2 (50%)		2 (50%)
4	QD 1 (41)		30 (73%)			6	
		9 (30%)		20 (67%)	5 (83%)		2 (33%)
5	QD 2 (17)		13 (76%)			20	
		8 (62%)		6 (46%)	18 (90%)		8 (40%)
6	QD 3C (10)		9 (90%)			2	
		1 (11%)		8 (89%)	2 (100%)		0 (0%)
7	QD 4 (16)		11 (69%)			0	
		1 (9%)		10 (91%)	0		0
8	QD 5 (21)		16 (76%)			1	
		9 (56%)		9 (56%)	1 (100%)		0 (0%)
9	QD 6 (6)		5 (83%)			10	
		4 (80%)		2 (40%)	9 (90%)		1 (10%)
Total			132 (72%)			51	
		32 (24%)		103 (78%)	45 (88%)		14 (27%)

^a^In brackets is the number of indicators within a quality dimension

QD 1 = Physical environment and equipment; QD 2 = Job expectations; QD 3A = Professional knowledge, skills and ethics (Integrated Management of Childhood Illnesses, IMCI); QD 3B = Professional knowledge, skills and ethics (Maternal health); QD 3C = Professional knowledge, skills and ethics (Fever); QD 3D = Professional knowledge, skills and ethics (HIV/AIDS and TB); QD 4 = Management and administration; QD 5 = Staff motivation; QD 6 = Client satisfaction

^b^For percentage figures the denominator is the number of indicators within a quality dimension

^c^For percentage figures the denominator is the number of indicators assigned to the same quality dimension

^d^For percentage figures the denominator is the number of indicators not assigned to the same quality dimension

## Discussion

### Appropriateness of the e-TIQH assessment tool to measure quality of care

#### Regression models versus in-depth interviews

Results from the regression models confirm previously reported preliminary findings [34]. Based on triangulation of data from regression models and in-depth interviews it could be concluded that quantitative and qualitative findings were overlapping and consistent. The only inconsistencies observed were the perceived gaps in health financing mechanisms, and a lack of medicines found in the qualitative but not quantitative results. The first concern was not captured by the e-TIQH assessment, since health financing was an issue beyond individual health facilities. The latter stood in contrast with the rather high scores in medicine availability in QD 4. This could partly be explained by the fact that only 16 essential medicines were tracked, and that medicine availability indicators were assessed using a more differentiated answer scale compared to all other indicators, where simple “yes/no/not applicable” answers were applied. Findings regarding differences in health facility level and owner categories were consistent between quantitative and qualitative findings. They were also in-line with what had been reported by other service assessments done in Tanzania [41, 42]. The fact that dispensaries were more likely to have insufficient and underqualified staff, and experienced more equipment and medicine stock outs, explained well why they were executing their mandate less well than health centers and hospitals. The importance of provider cadre for quality of care was also reported by others [43]. The problem of medicine and equipment availability at dispensary level was in-line with previous findings [41, 44, 45]. Importantly, the finding that dispensaries were given less priority by the council and other stakeholders may increase inequity in health since remote populations tend to be poorer and only have access to lowest level of care. For the private-not-for-profit sector, politeness of staff, external support as well as collaborations with the public sector was likely to have compensated certain deficits of the private sector and led to better overall performance compared to the private-for-profit sector. For the public and the private-not-for-profit sector the overall difference was small, and performance strongly varied between QDs. This was in-line with findings from other studies, which pointed out strengths and weaknesses of each sector [46–49]. Additionally, it has to be acknowledged that the assessments were mainly done by public employees and only by some representatives from the non-public sector [34]. Thus, there was a potential measurement bias, whereby public employees might have given better scores to health facilities of their own sector.

#### Quantitatively versus qualitatively ranking

Although our results showed good consistency, a comparison between the quantitatively and qualitatively generated health facility quality rankings revealed some limitations of quantitative measures. The results made clear that factors not directly related to quality of care (number of indicators assessed and average of answers given per indicator) could influence the assessment results. Addressing these factors would make the assessment technically more demanding, time-consuming and expensive, leading to decreased efficiency and feasibility during routine supportive supervision exercises. All of which can ultimately affect effectiveness. This therefore illustrated the constant trade-off between implementation feasibility, efficiency, effectiveness, validity, precision and acceptance of quality assessment measures.

#### Number and weights of indicators

By investigating the effect of changing the number and weights of indicators, we tried to assess how robust the e-TIQH assessment tool is in its ability to assign scores to health facilities and rank them accordingly. In terms of number of indicators, there is clearly a threshold above which neither score nor rank changes much anymore. The results showed that this number might have already been reached in the case of the e-TIQH assessment tool if the primary interest lies in the score and not the rank (e.g. if used for benchmarking purposes). Also, given their limited resources, providers and district authorities may find it easier to prioritize and address a smaller number of non-fulfilled indicators. Therefore, a set of few indicators, which are seen as most relevant for quality improvement, might lead to better results than a more comprehensive set of indicators.

The fact that indicators with high weights were a little more likely to be answered with “yes” than those with low weights showed that weights given to indicators during the e-TIQH development process reflected the priorities of the healthcare providers. However, results also revealed that weighting indicators only fine-tuned the scoring system and did not change scores or ranks drastically. Based on these findings, and considering the additional issues of design and analysis, it seems appropriate to recommend dropping the weighting. This would be in-line with a comparative analysis of selected health facility assessment tools which found that none of them used a weighting system [13].

#### Grouping of indicators

Based on a factor analysis we assessed the usefulness of grouping the indicators into the nine QDs and sub-QDs. The analysis confirmed that the factors reflected to a large extent the grouping done during the e-TIQH development process and therefore the grouping may be considered justifiable. Nevertheless, factor analysis also highlighted a couple of potential areas for improvement. Firstly, it suggested the subdivision of QD 4, whereby availability of medicines would be measured as a separate QD, while more general management and administration issues could be merged with other QDs. Secondly, factor analysis revealed that for the case of the e-TIQH assessment tool some indicators did not seem to be that relevant for predicting quality of care due to a similar strong association to another factor and a weak association to the factor the indicators were assigned to. Therefore, they could potentially be excluded. In particular client satisfaction appeared to have rather low relevance in predicting quality of primary healthcare. This finding was confirmed by the regression model, showing no significant difference in client satisfaction between health facility level and owner categories, despite the fact that the other scores showed clear differences. One reason why client satisfaction as it was captured in QD 6 did not reflect well the quality of health facilities, could be that the exit interview design had a courtesy bias (i.e. the patient not wanting to say anything negative about the facility). Courtesy bias has often been shown to be strong when interpreting perceived quality [13, 50, 51]. We tried to minimize the risk through rather objective indicators but it was certainly still influencing the respondent’s answers. Another reason could be that the patients simply could not judge the quality of care. A fair conclusion would thus be that client satisfaction is not a very good measure of quality of care, despite its apparent attractiveness. This is in-line with other findings [52–55]. However, qualitative data showed that assessing client satisfaction increased provider accountability and acceptance of the assessment within the community, and thus is still recommended to be considered when developing quality improvement initiatives [5].

### Application of the e-TIQH assessment tool

Overall, the results presented here together with previously reported findings [34] strongly suggested that the e-TIQH assessment tool, which focused on processes and structural adequacy of healthcare, is accurate enough to assess and monitor quality of primary healthcare for the purpose of routinely steering improvement measures. In practice, its ability to measure quality of care over time reflected a feasible approach to be used during supportive supervision and received great support from the CHMTs and health facilities staff [36]. However, the value of the e-TIQH assessment tool would need to be carefully reassessed if it were to be used outside its intended purpose. Potentially, it could be utilized for balanced score cards or benchmarking systems, as well as non-financial performance-based recognition initiatives [13, 28, 56–59]. Obviously, the accuracy of the assessment is crucially dependent on both the assessor and the health facility staff understanding the value of an objective evaluation, with the intent of improving the situation. Yet, there is a conflict of interest if this assessment tool would be used for some kind of performance-based payments as this might lead to adverse effects [60]. Our results showed that health facilities offering fewer services or having less staff could potentially be favored. Also, there could be an incentive to foster indicator driven improvements, although this would be less likely an issue due the holistic nature of the e-TIQH assessment tool [61–64]. Additionally, since the outcome of the assessment would have a financial value, there are legitimate concerns that providers could try to manipulate the assessment, whereas on the assessor’s side it is likely to augment corruption problems. Finally, due to its design and purpose the e-TIQH assessment tool in its current format is unlikely to be accurate enough for higher level of care, licensing or accreditation as well as providing evidence for national policy, planning or management decisions.

### Limitations of the study

It is recognized that well-trained assessors familiar with the context are key for the accuracy of the assessment and important to reduce measurement errors, especially when observing clinical consultations. For direct observations, it could not be excluded that there was a Hawthorne effect as suggested by others, although for this study the qualitative data could not confirm that [65–67]. Additionally, 21 health facilities could not be reached in at least one of the years due to their remote location. It has to be suspected that quality of care in such areas was below average. Thus, the missing data from these health facilities could have led to an overestimation of the average scores presented.

The paper did not elaborate on the differences between the six QD scores as this was discussed previously by Mboya et al. [34]. The present analysis did also not compare absolute values, time trends or differences between QDs with other quality of care measures. Further, the manuscript did not examine in all details time trends of quality scores or address the issue of how much the changes in quality of care could be attributed to the e-TIQH approach. These two points will however be investigated in a subsequent paper [35]. The study did additionally not demonstrate how the approach was able to increase more generally the feasibility of routine supportive supervision, but this was shown elsewhere [36]. Finally, none of the studies examined the effects of the e-TIQH assessment tool or improvements in quality of care on changes in health outcomes. Hence, the proof that improved processes lead to improved health outcomes is still outstanding. This could be subject of further research, for example through linking community health data with health facility data.

## Conclusions

Despite the lack of standards regarding how to best measure quality of care, the results presented here, coming from a range of different methods, suggested that for the purpose of routinely steering improvement measures at local level the e-TIQH assessment tool was able to accurately assess and monitor quality of primary healthcare. Focusing the quality assessment on processes and structural adequacy of healthcare was an appropriate approach for the assessment’s intended purpose, and a unique key feature of the e-TIQH assessment tool. Thus, the e-TIQH assessment tool demonstrated a feasible option for routine quality measures of primary healthcare of different health facility level and owner categories in Tanzania. The findings, combined with the more operational results of the companion papers [35, 36], created a solid foundation for an approach that could lastingly improve services for patients attending primary healthcare. Finally, the expanded use of the e-TIQH assessment tool, for example for performance-based payment schemes, accreditation and other systematic evaluations of healthcare quality, should be considered carefully because of the risk of bias and adverse effects.

## Abbreviations

AIC: Akaike Information Criterion
CHMT: Council Health Management Team
CHSB: Council Health Service Board
DC: District Council
e-TIQH: electronic Tool to Improve Quality of Healthcare
IMCI: Integrated Management of Childhood Illness
LMIC: Low and Middle-Income Countries
MC: Municipal Council
NIMR: National Institute for Medical Research
PPP: Private Public Partnership
QD: Quality dimension
UHC: Universal Health Coverage
